# Evaluation of the Repolished Surface Properties of a Resin Composite Employing Structural Coloration Technology

**DOI:** 10.3390/ma14237280

**Published:** 2021-11-28

**Authors:** Mayumi Maesako, Takafumi Kishimoto, Shigetaka Tomoda, Taku Horie, Mitsuyoshi Yamada, Rika Iwawaki, Yukari Odagiri, Keiko Sakuma, Kazuho Inoue, Ayumi Takeguchi, Miki Suzuki, Akio Mitani, Morioki Fujitani

**Affiliations:** 1 Department of Operative Dentistry, School of Dentistry, Aichi Gakuin University, Nagoya 464-8651, Japan; ag183d11@dpc.agu.ac.jp (M.M.); taka-ki@dpc.agu.ac.jp (T.K.); lifedoor@dpc.agu.ac.jp (T.H.); mitsuyos@dpc.agu.ac.jp (M.Y.); iwawaki@dpc.agu.ac.jp (R.I.); mado1209@dpc.agu.ac.jp (Y.O.); virgo@dpc.agu.ac.jp (K.S.); kazuho@dpc.agu.ac.jp (K.I.); Ayumiiii@dpc.agu.ac.jp (A.T.); mikis@dpc.agu.ac.jp (M.S.); morioki@dpc.agu.ac.jp (M.F.); 2 Department of Periodontology, School of Dentistry, Aichi Gakuin University, Nagoya 464-8651, Japan; minita@dpc.agu.ac.jp

**Keywords:** resin composite, structural coloration technology, repolishing, surface property, alkaline degradation

## Abstract

Resin composites employing structural coloration have recently been developed. These resins match to various tooth shades despite being a single paste. To accomplish this, the filler and base resin are tightly bonded, which is thought to provide excellent discoloration resistance. Here, we investigated the surface properties of one of these resins, including the discoloration of the repolished surface. We developed an innovative in vitro method to adjust the repolished surface, in which structural degradation is removed according to scanning electron microscopy (SEM) observation rather than by the naked eye. The resin samples (20 mm (length) × 10 mm (width) × 4 mm (depth)) were manufactured using this resin material. After accelerated aging of the resin by alkaline degradation, the resin was repolished and the discoloration (ΔE*ab), surface roughness (the arithmetic mean roughness (Ra)), and glossiness (the 60° specular) were measured. SEM observation showed that the appearance of the bond between the organic composite filler and base resin on the repolished surface was different from that on the mirror-polished surface. This revealed that according to our in vitro method it was difficult to make the repolished surface structurally identical to the mirror-polished surface. Among the properties of the repolished surface, the degree of discoloration did not change despite the rougher and less glossy surface. It can be concluded that the factors that induce discoloration in this resin composite are independent of the surface roughness and glossiness.

## 1. Introduction

Dental caries is the main dental disease, and the treatment of dental caries is important to maintain the oral function and esthetics of patients. In dental practice, dental materials are essential for dental treatments, including caries treatment. In particular, resin composites are typical dental materials used for dental caries treatment, and they have been steadily improved, developed, and marketed. The shade of the resin composite that fills the cavity is very important from an esthetic point of view, and it should match the shade of the tooth around the cavity.

Recently, a new resin composite called OMNICHROMA (OC, Tokuyama Dental, Tokyo, Japan) has been developed that matches to a variety of tooth shades despite being a single paste. The basic composition of this resin, a filler and a base resin, is the same as that of conventional resins, but a new technology called structural coloration has been introduced [1]. Structural coloration is a phenomenon where the substance has no pigment but is colored by the reflection of light based on a nano structure (e.g., thin-films, diffraction gratings, or photonic crystals) below the wavelength of visible light [2]. This newly developed resin contains uniform spherical supra-nanoparticles that are 260 nm in diameter. It is assumed that the size of the nanostructure enhances a particular color tone. This size of the nanoparticle filler generates red to yellow colors related to the color of human teeth [1,3,4,5]. This filler also provides a blending effect of the reflected light from the resin surface and diffuses the reflected light from the filler and base resin contained in the resin composite and the background tooth color. This unique and interesting technology makes it suitable for various tooth shades. It has also been reported that resin composites containing uniform spherical filler particles (260 nm diameter) exhibit structural color and have good color compatibility with denture teeth of various shades [3].

Some conventional resin composites do not have structural coloration even though they contain fillers of a similar size and shape. Therefore, in OC, to achieve high color compatibility, the basic composition of the filler and base resin, as well as the silanization technology for both, should be improved and enhanced. However, no detailed studies have been reported. To clarify the factors that cause this resin to match tooth shades, we have compared the particle size, distribution, and density of the filler, and the junction between the filler and base resin with conventional composite resins using the alkaline-degradation test [6,7,8,9,10,11]. The results suggested that to enable structural coloration, the tight bonding of the spherical organic filler particles and base resin is essential and light incident on the resin composite must pass through the filler–base resin junction interface without reflection or interference [11].

Discoloration is one of the most common causes of the aging degradation of resin composites, and the junction between the filler and base resin affects discoloration. This is due to detachment/dropout of the filler caused by hydrolysis of the siloxane bond and the penetration of pigment [12,13,14,15]. In a clinical report, Peumans et al. reported discoloration in 42% of resin composite cases in their 5-year clinical follow-up report [16]. Ferrari et al. found that 32.5% of cases showed discoloration and 15% showed discoloration that required repolishing in their 5-year clinical follow-up report [17]. Shimizu et al. reported that 5% of cases showed discoloration that required refurbishment at the 10-year clinical follow-up [18]. Although repolishing discoloration is an indispensable procedure to maintain the esthetic quality of the restored resin, there have been no reports on resin materials employing structural coloration in which the filler and base resin are tightly bonded.

Test methods for reproducing aging degradation in the laboratory have been reported, such as the long-term water aging test [12,13,15,19,20,21], immersion in food simulating liquid test [14,22,23], and weathering light test [24]. Among these methods, the alkaline-degradation test has often been used to simulate accelerated degradation of the tight bonding of the filler and base resin.

In this study, we investigated the discoloration, surface roughness, and glossiness of the repolished surface of OC after mimicking aging in vitro by the alkaline-degradation test and compared the results with those before aging degradation. The hypothesis was that the repolished surface after the aging degradation of the resin material employing structural coloration would reproduce the surface properties before the aging degradation.

## 2. Materials and Methods

### 2.1. Material

The materials used are shown in Table 1. The components of OC (Tokuyama Dental, Tokyo, Japan), a resin composite employing structural coloration technology, are shown in Table 2.

### 2.2. Preparation of the Mirror-Polished Samples and Measurement of the Surface Properties

**Figure 1:**
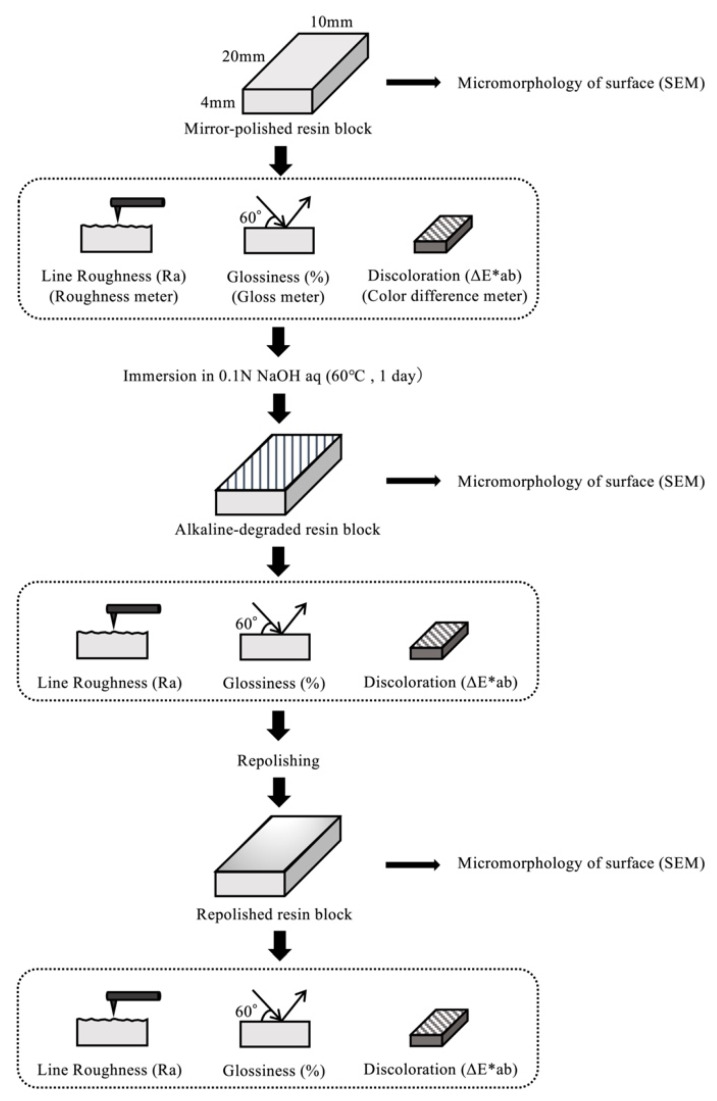
Experimental procedures.

**Figure 2:**
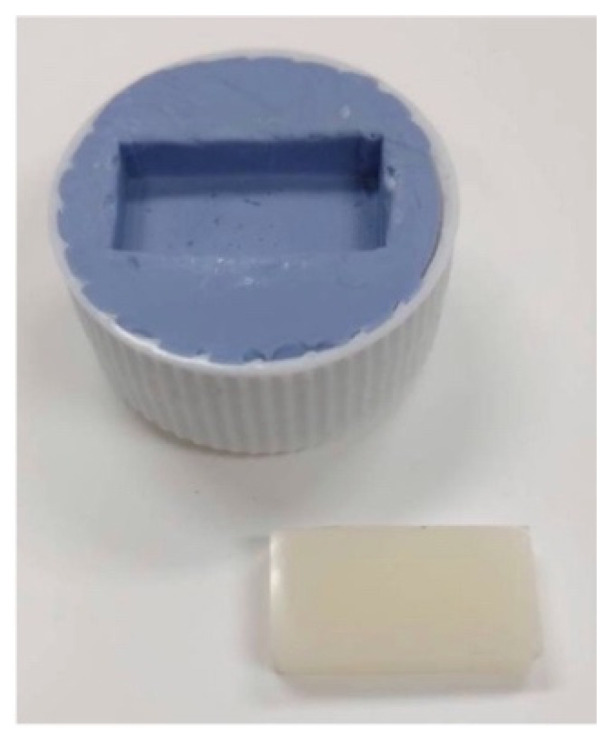
Silicone mold (20 mm (length) × 10 mm (width) × 4 mm (depth)) and resin block.

The experimental procedure is shown in Figure 1. This composite resin was placed in layers into the concave silicone mold (20 mm (length) × 10 mm (width) × 4 mm (depth) Figure 2) and each layer was light cured using a light-curing unit (VALO Curing Light, Ultradent Japan, Tokyo, Japan) (1000 mW/cm^2^). To obtain an amply polymerized flat surface, the top of the resin block was light cured for 60 s as it was pressed with a glass slide through a polyethylene film. The polymerization was carried out under an anaerobic environment to remove the unpolymerized layer. These samples, an anaerobic agent (disposable O_2_ absorbing and CO_2_ generating agent, Mitsubishi Gas Chemical Company, Tokyo, Japan), and an anaerobic indicator (oxygen indicator, Mitsubishi Gas Chemical Company) were placed in an anaerobic culture jar (2.5 L standard square jar, Mitsubishi Gas Chemical Company) and kept in a thermostatic bath (37 °C) for 1 day.

One surface (20 mm × 10 mm) of the resin block sample was sequentially finished with #800, #1200, #1500, and #2000 water-resistant silicone papers using an automatic rotary polishing machine (Ecomet 3000, Buehler, Lake Bluff, IL, USA) under continuous water injection. The mirror-polished surfaces were prepared using 1.0 µm and 0.3 µm of aluminum oxide powders and polishing buffs. Between each step, ultrasonic cleaning with distilled water was performed (three times for 5 min). Various surface properties were measured, such as the discoloration, line roughness, and glossiness (*n* = 15).

#### 2.2.1. Discoloration Test (Color Difference)

**Figure 3:**
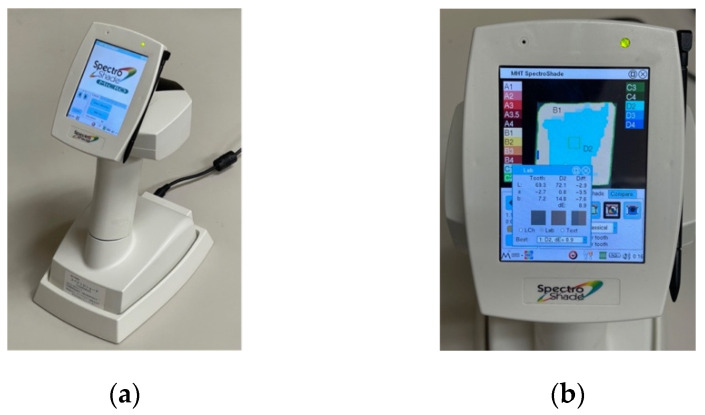
(**a**) Dental spectrophotometer and (**b**) its measurement screen.

The mirror-polished samples were immersed for 7 days in tea solution adjusted to 37 °C prepared by boiling three tea bags (Nitto tea 2 g, Mitsui Norin, Tokyo, Japan) in 250 mL of distilled water for 5 min. The color of the resin surface before and after immersion was measured at three locations per sample on a standard black plate using a dental spectrophotometer (Spectro Shade, Spectro Shade USA, Oxnard, CA, USA), and the color difference (ΔE*ab) was calculated from the measured values (Figure 3a,b). The average value of the color difference was used as the discoloration of the samples.

ΔE*ab = ((ΔL*)^2^ + (Δa*)^2^ + (Δb*)^2^)^1/2^ (1)

#### 2.2.2. Line Roughness Test

**Figure 4:**
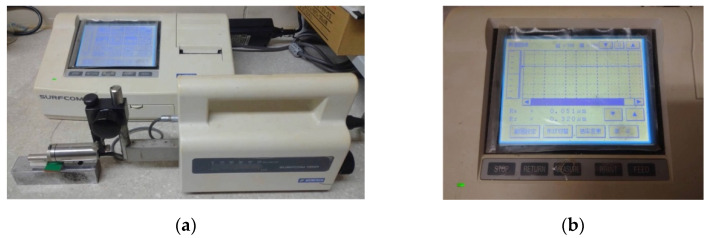
(**a**) Handheld glossiness meter and (**b**) its measurement screen.

The arithmetic mean roughness (Ra) was determined using a surface roughness meter (Surfcom 130A, Tokyo Seimitsu, Tokyo, Japan) at a feed rate of 0.15 mm/s (Figure 4a,b). Three measurements were taken near the center of each sample, and the average value was used as the line roughness of the sample.

#### 2.2.3. Glossiness Test

**Figure 5:**
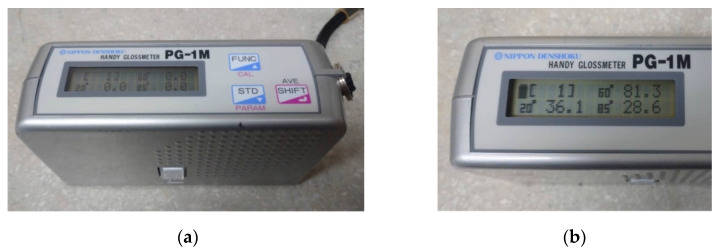
(**a**) Surface roughness meter and (**b**) its measurement screen.

A handheld glossiness meter (PG-1M, Nippon Denshoku, Tokyo, Japan) was used to measure the 60° specular glossiness according to Japanese Industrial Standards (Figure 5a,b). Five measurements were taken around the center of each sample, and the average value was used as the glossiness of the sample.

### 2.3. Preparation of the Alkaline-Degraded Samples and Measurement of the Surface Properties

The samples after the measurements were immersed in 0.1 N NaOH aqueous solution (60 °C, pH 12.7) for 1 day to accelerate degradation. After ultrasonic cleaning (three times for 5 min), each surface property was measured in the same way as for the mirror-polished samples.

### 2.4. Preparation of the Repolished Samples and Measurement of the Surface Properties

The alkaline-degraded sample was cut in the longitudinal direction so that the cross-section could be observed. The superficial layer degraded by alkaline immersion (degraded layer) was ground off from the sample surface while observing the cross-section by scanning electron microscopy (SEM, VE-9800, Keyence, Osaka, Japan).

**Figure 6:**
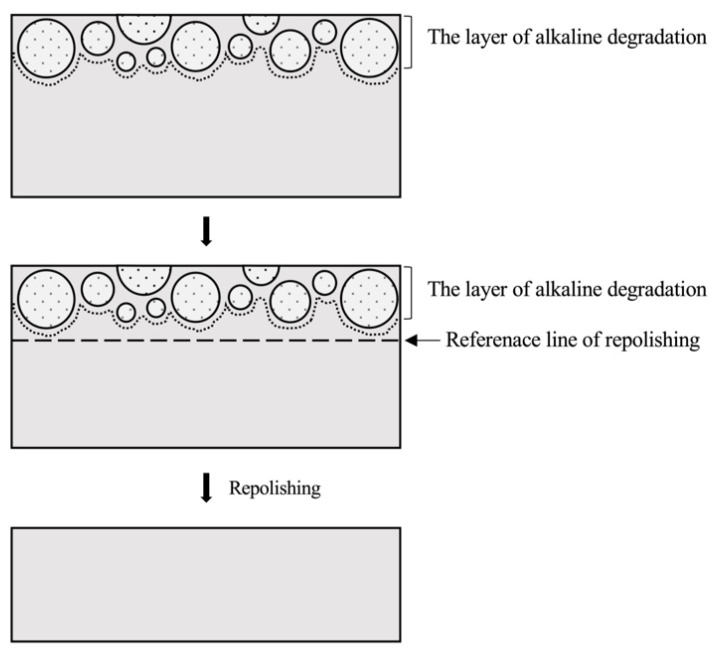
Schematic diagram of the repolished surface preparation procedure.

After removing the degraded layer, the surface was finally polished with 0.3 μm of aluminum oxide, which was used as the repolished sample (Figure 6). Each surface property was measured in the same way as for the mirror-polished sample.

### 2.5. Morphology Observation (SEM)

The mirror-polished, alkaline-degraded, and repolished samples were separately prepared for observation in the same way, as described above. After ultrasonic cleaning (three times for 5 min), the surface of each sample was sputtered with 10 nm-thick gold (MSP-1S, Vacuum Device, Ibaraki, Japan) and observed by SEM.

### 2.6. Statistical Analysis

The differences between the line roughness, glossiness, and discoloration of the mirror-polished, alkaline-degraded, and refurbished samples were assessed with one-way ANOVA. When the results were statistically significant (*p* < 0.05), Bonferroni’s test was used in the post-hoc analysis (α = 0.001). The data were processed using SPSS.

## 3. Results

### 3.1. Effect of Alkaline Degradation and Repolished on the Resin Surface Properties

#### 3.1.1. Color Difference

**Figure 7:**
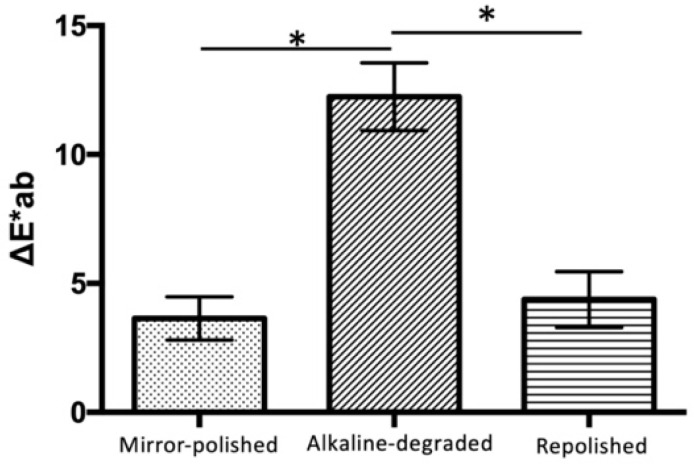
Color difference after the discoloration test. The bars (*) indicate the statistical difference (Bonferroni’s test, *p* < 0.001, *n* = 15).

The color differences of the mirror-polished, alkaline-degraded, and repolished samples after the discoloration tests were 3.64 ± 0.81 ΔE*ab, 12.25 ± 1.27 ΔE*ab, and 4.38 ± 1.04 ΔE*ab, respectively (Figure 7, Table 3). The alkaline-degraded samples showed a significantly higher discoloration score than the mirror-polished and repolished samples (*p* < 0.001). No significant difference was observed between the mirror-polished and repolished samples.

#### 3.1.2. Line Roughness

**Figure 8:**
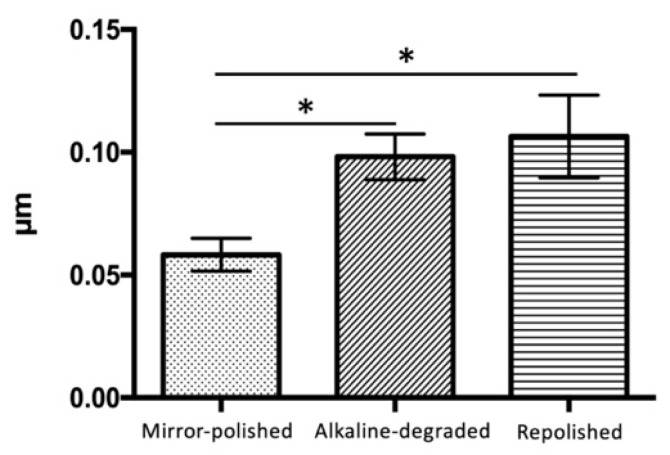
Line roughness. The bars (*) indicate the statistical difference (Bonferroni’s test, *p* < 0.001, *n* = 15).

The line roughness values of the mirror-polished, alkaline-degraded, and repolished samples were 0.058 ± 0.006 μm, 0.098 ± 0.009 μm, and 0.106 ± 0.016 μm, respectively (Figure 8, Table 3). The alkaline-degraded and repolished samples showed higher roughness values than the mirror-polished samples (*p* < 0.001). No significant difference was observed between the alkaline-degraded and repolished samples.

#### 3.1.3. Glossiness

**Figure 9:**
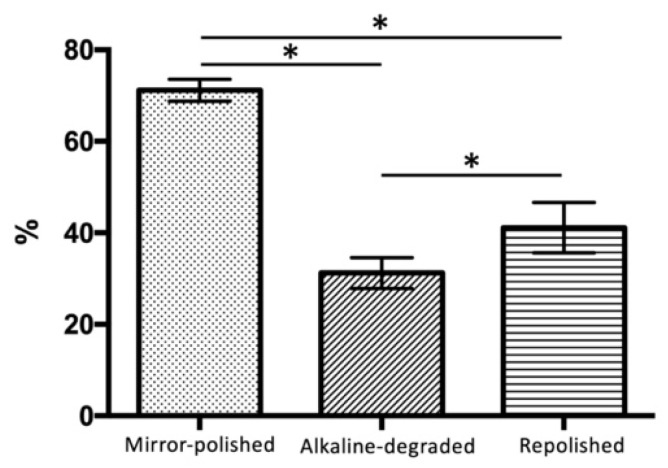
Glossiness. The bars (*) indicate the statistical difference (Bonferroni’s test, *p* < 0.001, *n* = 15).

The glossiness values of the mirror-polished, alkaline-degraded, and repolished samples were 71.17 ± 2.29%, 31.22 ± 3.26%, and 41.13 ± 5.34%, respectively (Figure 9, Table 3). The glossiness values of the alkaline-degraded and repolished samples were significantly lower than that of the mirror-polished samples (*p* < 0.001). Repolishing significantly recovered the glossiness of the alkaline-degraded samples (*p* < 0.001).

### 3.2. Effect of Alkaline Degradation and Repolished on the Resin Surface Morphology

**Figure 10:**
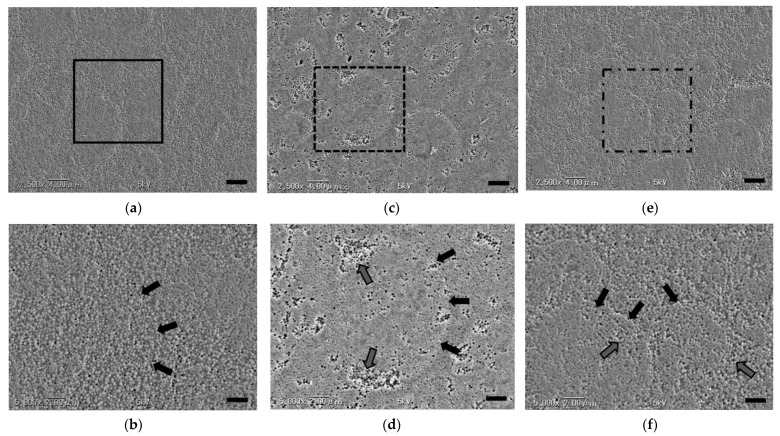
Representative SEM images of the various samples. (**a**) Mirror-polished sample (bar = 4.0 µm). (**b**) Higher magnification image of the square in (**a**) (bar = 2.0 µm). The black arrows indicate the filler and base resin are tightly bonded. (**c**) Alkaline-degraded sample (bar = 4.0 µm). (**d**) Higher magnification image of the dashed square in (**c**) (bar = 2.0 µm). The black arrows indicate the boundaries between the spherical organic filler particles and base resin. The gray arrows indicate voids due to dropout of inorganic filler particles. (**e**) Repolished sample (bar = 4.0 µm). (**f**) Higher magnification image of the dot-dashed square in (**e**) (bar = 2.0 µm). The black arrows indicate the boundaries of the spherical organic filler particles. The gray arrows indicate voids due to dropout of inorganic filler particles.

The morphology of each of the sample surfaces from SEM analysis is shown in Figure 10. Organic composite filler particles of various sizes, circular inorganic filler particles, and base resin particles were observed in the mirror-polished samples. Uniformly sized spherical inorganic filler particles were homogenously dispersed in the organic composite filler and base resin at about the same density. There were no gaps at the boundaries between the various fillers and the base resin, and a tight bond was maintained (Figure 10a,b). In the alkaline-degraded samples, the degrees of degradation of the inorganic filler and base resin, as well as the inorganic filler and base resin almost at the center of the organic composite filler, were almost the same. Around the superficial layer of the organic composite filler, a degraded layer that clearly delineated the boundary with the base resin was observed, and many voids due to drop-out of the inorganic filler were observed (Figure 10c,d). In the repolished samples, the tight bond between the organic composite filler and base resin was clearer (Figure 10e,f). However, more inorganic filler was observed to have dropped out than from the mirror-polished samples (Figure 10b,f).

## 4. Discussion

We found that the newly developed resin composite OC, in which structural coloration technology is introduced, has the same basic composition as conventional resin composites, but the siloxane bonding technology at the junction of the filler and base resin is dramatically improved [11]. In particular, the bonding between the organic filler and base resin was found to be important. If the junction between the filler and base resin is good, there will be no peeling of the filler due to hydrolysis of the siloxane bond and no dye penetration into the filler drop-out areas [12,13,14,15]. From this, we hypothesized that OC would be less prone to discoloration and the repolished surface after aging would show less discoloration and other surface property (roughness and glossiness) changes. There have been no reports on the properties of repolished surfaces after aging in vitro. The results of this study show that the degree of discoloration of the repolished surface of the OC did not change compared with the surface before aging, but the roughness and glossiness changed.

The bonding condition between the filler and base resin has a significant influence on the surface properties [12,13,14,15,25]. As the surface properties of resin composites degrade over time, discoloration, surface coloration, wear, and loss of glossiness occur, resulting in a significant loss of esthetics. As mentioned in Section 1, discoloration associated with the degradation of resin composites has been reported in clinical practice. Although the age of each report is different and there are differences in the resin composite materials owing to technological innovation, discoloration associated with aging degradation is a major problem with restored resin composites [16,17,18]. In addition, very few studies have investigated discoloration associated with aging degradation in vitro, and there are still many unanswered questions. Therefore, it is necessary to clarify which part of the aged resin is preferentially destroyed. We believe that the cause of the surface degradation is the fractured part of the aged resin.

We chose the alkaline-degradation test to reproduce the aging-degradation process in vitro. This alkaline-degradation test is a type of accelerated aging test. By immersing resin composites in an alkaline solution with a concentration that is impossible to apply in the oral cavity, aging degradation of the resin composites can be simulated in a short time. This can reveal areas of weakness in the resin structure that occur with aging.

SEM images of the alkaline-degraded OC surface showed that many of the inorganic filler particles dropped out (indicated by the gray arrows in Figure 10d). However, the organic composite filler and base resin, which are difficult to bond with siloxane [20,25], as previously reported by the authors (indicated by the black arrows in Figure 10b,d,f) [11], were confirmed to be tightly bonded without any detachment even in an alkaline environment. The color differences of the alkaline-degraded samples showed a significantly higher discoloration score than the other samples. The cause of the discoloration is suggested to be the stagnation of the tea stain in the voids owing to drop-out of the inorganic filler particles. These voids also affect the roughness and glossiness. In this study, it is considered that when the resin composite employing structural coloration technology is degraded with aging, the inorganic filler will first drop-out, which will affect various surface properties.

If discoloration of the resin composite is clinically observed, the discolored area should be removed and repolished. In general, the need for repolishing is judged only by the color difference determined by the clinician’s naked eye. The National Bureau of Standards unit value for evaluating the color difference with the naked eye is a six-level evaluation classification of the color difference based on the value of 0.92 × ΔE*ab [26,27,28]. However, this evaluation method has a wide range of unit values, and it is difficult to eliminate discolored areas in in vitro studies under these conditions. Therefore, we focused on the structure of the resin composite material and devised a highly reproducible method for preparing repolished samples in vitro. As shown in the procedure in Figure 6, after the alkaline-degradation test, all the samples were removed while checking the cross-sectional SEM images. This series of preparation steps was followed to ensure that each sample had the same repolishing conditions.

By SEM observation, there was no significant decrease in the inorganic filler in the repolished sample, confirming that the degraded layer was sufficiently removed by repolishing. The newly devised method for the preparation of repolished samples was found to be useful. However, more inorganic filler dropped out than for the mirror-polished samples (indicated by the gray arrows in Figure 10d,f). These results suggest that it is difficult to make the repolished OC surface structurally identical to the mirror-polished surface before degradation using the present preparation method. The limitations of this method, which adjusts for structural observations in only two dimensions, were also specified. In addition, surface analysis using EDS (energy dispersive X-ray spectroscopy) or XPS (X-ray photoelectron spectroscopy) should be conducted to study the surface properties in detail.

From the discoloration test, there was no significant difference in the discoloration resistance between the mirror-polished and repolished samples (Figure 7). This suggests that the surface properties before and after the effects of degradation were similar. Using the reported preparation method, it was clarified that no discoloration occurred if there was no dropout of inorganic filler particles.

In terms of the line roughness, there was a significant difference between the mirror-polished and repolished samples (Figure 8). It was confirmed that the surface was rough even after repolishing. The reason why there was no significant difference between the repolished and alkaline-degraded samples was that dropout of 260 nm inorganic filler particles had no effect on the line roughness. In terms of the glossiness, there were significant differences among the various samples (Figure 9). It was confirmed that the glossiness level was low even after repolishing. The difference in the appearance of the junction between the organic composite filler and base resin observed by SEM was considered to affect the surface roughness and positive reflectance.

The surface of the repolished OC sample was rough and had low glossiness, although the discoloration resistance was good. Based on the results of this study, the hypothesis was partially rejected. In other words, the results suggest that in clinical cases of discoloration of OC, there is no change in the surface roughness or glossiness, that is, there is no reversion even after repolishing to remove discoloration by the naked eye. Moreover, it is presumed that discoloration of the resin composite is independent of the surface roughness and/or glossiness [29]. This interesting finding is supported by reports that resin discoloration is caused by stagnation of the stain at the filler–base resin junction and/or rough surface [12,13,14,15].

A limitation of this study is that it did not compare each experiment with a conventional resin composite for consideration of only the repolished surface. To clarify whether the phenomenon observed in this study is unique to resin composites employing structural coloration technology, a comparative study is necessary. In addition, to further clarify the relationship between the degree of discoloration and each of these factors, additional experiments on the wettability and surface free energy need to be performed [30].

## 5. Conclusions and Implications

The degree of discoloration is generally related to the surface roughness of the composite material. Interestingly, from our results, the degree of discoloration of the repolished OC resin composite might be the same as that of the mirror-polished composite, even though the surface is rougher and less glossy. Further investigations are needed for confirmation.

The resin composite employing structural coloration evaluated in this study showed excellent discoloration resistance after repolishing because of the tight bond between the organic composite filler and base resin. This suggests that predictable and reliable resin composite restoration is clinically feasible. Furthermore, this study will provide information for the development of new composite materials.

## Figures and Tables

**Table 1:** Material used.

Material	Product Name	Manufacturer
Resin composite	OMNICHROMA	Tokuyama Dental Corporation (Tokyo, Japan)
Silicone mold	Blue eco (Base + Catalyst)	DETAX GmbH & Co. KG (Ettlingen, Germany)
Polyethylene film	Matrix tape	3M Japan Limited (Tokyo, Japan)
Glass slide	ASLAB Slide Glass	AS ONE Corporation (Osaka, Japan)
Anaerobic agent	Disposable O_2_ absorbing and CO_2_ generating agent	Mitsubishi Gas Chemical Company (Tokyo, Japan)
Anaerobic indicator	Oxygen indicator	Mitsubishi Gas Chemical Company (Tokyo, Japan)
Anaerobic culture jar	2.5 L standard square jar	Mitsubishi Gas Chemical Company (Tokyo, Japan)
Silicone paper	Waterproof silicone carbide paper disc	Refine Tec Ltd. (Yokohama, Japan)
Aluminum oxide powder	Almina polishing compound	Refine Tec Ltd. (Yokohama, Japan)
Buff	Suede cloth	Refine Tec Ltd. (Yokohama, Japan)
Tea	Nitto tea	Mitsui Norin (Tokyo, Japan)
NaOH aqueous solution	2 mol/L Sodium hydroxide solution (2N)	KANTO CHEMICAL CO., INC. (Tokyo, Japan)

**Table 2:** Components of OMNICHROMA.

Brand Name	Resin Type	Filler Type	Filler Size	Filler Contents (Weight %)	Base Resin	Manufacturer	Lot. No	Code
OMNICHROMA	Paste	Uniform sized supra-nano spherical filler Organic filler	φ260 nm	79	UDMA TEGDMA	Tokuyama Dental	0343	OC

**Table 3:** Result of measurements.

	Color Difference (ΔE*ab)	Line Roughness (μm)	Glossiness (%)
Mirror-polished	3.64 (0.81) ^a^	12.25 (1.27) ^ab^	4.38 (1.04) ^b^
Alkaline-degraded	0.058 (0.006) ^cd^	0.098 (0.009) ^c^	0.106 (0.016) ^d^
Repolished	71.17 (2.29) ^ef^	31.22 (3.26) ^eg^	41.13 (5.34) ^fg^

The same superscript letters indicate significant difference (Bonferroni’s test, *p* < 0.001, *n* = 15).

## Data Availability

The data presented in this study are available on request from the corresponding author.
